# Association between Dietary Behavior and Mental Health in Adolescents from Multicultural and Non-Multicultural Families in Korea

**DOI:** 10.3390/nu18111686

**Published:** 2026-05-25

**Authors:** Jeong-Hwa Choi, Young-Ran Heo

**Affiliations:** 1 Department of Food Science and Nutrition, Keimyung University, Daegu 42601, Republic of Korea; jhchoi@kmu.ac.kr; 2 Department of Food and Nutrition, Research Institute for Human Ecology, Chonnam National University, Gwangju 61186, Republic of Korea

**Keywords:** adolescents, dietary behavior, disparity, generalized anxiety disorder, multicultural family, suicidal behavior

## Abstract

**Background/Objectives:** The growing population of multicultural adolescents (MCAs) has become a vital focus for national health policy. Despite their increasing numbers, MCAs often encounter unique socioeconomic challenges and dietary issues that may heighten mental health vulnerabilities. This study aimed to assess the dietary behaviors of MCA and non-MCA and to explore the association between these behaviors and mental health outcomes, specifically generalized anxiety disorder (GAD) and severity of suicidal behavior. **Methods:** Using data from the 2024 Korea Youth Risk Behavior Survey, we analyzed the dietary behaviors of 44,796 adolescents, focusing on five key areas: breakfast consumption, fruit intake, caffeine drinks, sweetened beverages, and fast food. We also calculated a composite dietary behavior score and assessed mental health using the GAD-7 and a three-component suicidal behavior scale (including ideation, planning, and attempts). **Results:** MCAs experienced significant disparities in socioeconomic status and had a notably higher prevalence of suicide attempts compared to non-MCAs (*p* = 0.0107). In both groups, poorer dietary behaviors were linked to an increased likelihood of GAD and greater severity of suicidal behavior (*p_trend_* < 0.05). This association with suicidal behavior severity was particularly pronounced in MCA (*p_interaction_* = 0.0358). **Conclusions:** Dietary behavior is significantly associated with mental health issues among Korean adolescents. Given the vulnerabilities faced by MCA, it is essential to implement multifaceted policy support and targeted dietary interventions to improve outcomes for this population.

## 1. Introduction

Adolescence is marked by significant physical and psychological changes. Adolescents undergo pubertal development, and their brains also experience major neural reorganizations [1,2]. The World Health Organization reports that one in seven adolescents suffers from a mental health disorder [3]. If these unstable psychological conditions are not identified and treated promptly, the resulting physical and mental health issues may persist into adulthood, leading to severe outcomes such as generalized anxiety disorder (GAD) and suicide [3]. Approximately 6.5% of children and adolescents worldwide are affected by GAD. Furthermore, suicide is the second leading cause of death among adolescents in the United States [4] and accounts for over 50% of teenage deaths in South Korea [5]. Various factors influence these mental health challenges during adolescence, including family dynamics, peer relationships, socioeconomic status, gender, ethnicity, and multicultural background [4,6].

In 2023, foreign residents made up about 4.8% of South Korea’s total population, and the proportion of newborns from multicultural backgrounds exceeded 5%. Consequently, the percentage of adolescents from multicultural families (MCA) is increasing. Although they constituted only 3.5% of the total student population in 2023, this represented a 7.4% rise from 2022 [7,8]. Given the significant decline in the overall student population due to South Korea’s low birth rate [9], addressing the growth of the MCA population is crucial. Therefore, the development and implementation of comprehensive multicultural policies are essential. In terms of mental health, adolescents from multicultural backgrounds are reported to be more vulnerable to physical and psychological issues, such as depression, anxiety, stress, eating disorders, and body image distortion. This vulnerability arises from living in environments where parents have different nationalities and diverse cultures coexist. Earlier studies on MCA individuals in Korea found no significant difference in the prevalence of GAD; however, these individuals exhibited higher rates of depression [10] and were more likely to consider or engage in suicidal behaviors compared to their non-MCA peers [11].

Recent studies have highlighted the associations between dietary habits, nutritional status, and mental health. Dietary factors have been associated with mental well-being and may contribute to reducing the risk of depression [12]. In contrast, poor dietary quality has been associated with psychological distress, including depressive symptoms, perceived stress, and suicidal ideation [13]. Moreover, consumption of junk food and high levels of simple sugars has been linked to an increased risk of mental health issues in adolescents, as shown by a meta-analysis [14] and supporting prospective evidence [15]. A meta-analysis also indicated that high-quality diets rich in fruits, vegetables, whole grains, and lean meats are associated with a lower risk of depressive symptoms [16]. Longitudinal studies suggest that a sufficient intake of fruit and adherence to healthy dietary patterns may lower the incidence of depression and anxiety [17,18], with consistent findings observed in children [19]. Collectively, these findings suggest that healthy dietary habits are associated with a lower risk of mental health issues, including GAD and suicide-related behaviors. However, numerous issues have been identified in the dietary habits of contemporary adolescents. In South Korea, adolescents are increasingly skipping breakfast, consuming insufficient fruits and vegetables, and turning to high-sugar foods and fast food. Since dietary habits formed during adolescence can persist into adulthood and significantly influence long-term health outcomes, it is vital to promote healthy eating habits while considering the socio-environmental factors at play.

For MCA, additional socio-cultural and economic factors further shape their eating behaviors. Notably, compared with non-MCAs, MCAs tend to consume fruits and vegetables less frequently [20,21] and display disparities in overall dietary habits and health status [22]. Although research has found no distinct association between fruit intake and mental health experiences specifically among MCAs [21], healthy dietary habits are generally associated with positive mental health in the broader adolescent population [23]. Therefore, it is essential to explore the relationship between dietary behavior and severe mental health issues, particularly anxiety and suicidal behavior, while considering the unique characteristics of multicultural backgrounds.

This study aimed to examine the association between dietary behavior and mental health—specifically GAD and suicidal behaviors—among adolescents with and without MCAs and non-MCAs. To achieve this, data from the 20th Korea Youth Risk Behavior Survey (KYRBS 2024), a recent, large-scale, nationally representative survey of South Korean adolescents, were analyzed.

## 2. Subjects and Methods

### 2.1. Cohort Description

KYRBS is a government-approved statistical survey conducted by the Korea Disease Control and Prevention Agency (KDCA, approval No. 117058). The survey examines various health behavior indicators among South Korean adolescents, including dietary habits, mental health, tobacco and alcohol consumption, and mobile device usage, among others. The survey is administered as a self-reported online questionnaire to students from the first year of middle school to the third year of high school, ensuring complete anonymity. For this study, data from the 20th KYRBS (2024) were obtained and analyzed following the official procedures outlined on the KDCA website. The 20th survey targeted a total of 800 schools, comprising 400 middle schools and 400 high schools. The final participant count included 54,652 students from 799 schools (400 middle schools and 399 high schools), resulting in a student-level response rate of 94.9%. This study received approval from the Institutional Review Board (IRB, No. 1040198-231006-HR-145-01).

### 2.2. Study Population and Classification of Multicultural Background

Among the 54,653 participants in the raw data of the 20th KYRBS (2024), those with missing information about general characteristics, parental nationality, mental health-related variables, or dietary habits were excluded (*n* = 9857). Consequently, 44,796 participants were included in the final analysis. For this study, students from multicultural families were defined based on survey items regarding parental nationality: Was your father/mother born in Korea? Students who answered yes for both parents were classified as the non-multicultural group, while those who answered no for at least one parent were classified as the multicultural group.

### 2.3. General Characteristics

To identify the general characteristics of the study participants, the following information was analyzed: gender, age, body mass index (BMI), school type, grade, city type, household economic status, living arrangement, smartphone use (on weekdays and weekends), and subjective health status. BMI was calculated by dividing body weight in kilograms by the square of height in meters (kg/m^2^). School type was classified as either middle school or high school, while city type was categorized into rural areas (county), small-to-medium cities (city), and large metropolitan areas (metropolitan city). Household economic status was divided into four levels: high, mid-high, middle, and low. Living arrangements were categorized as either living with family/relatives (cohabitation) or living separately (alone). Subjective health status was assessed using four categories: very healthy, healthy, average, and unhealthy. For smartphone usage, participants were first categorized based on whether they used a smartphone (yes/no) on weekdays and weekends, followed by measuring the actual duration of use in minutes for further analysis.

### 2.4. Assessment of Dietary Behaviors and Score

To assess the dietary status of the participants, five items from the KYRBS were utilized: frequency of breakfast consumption, fruit intake, high-caffeine energy drink consumption, sugar-sweetened beverage intake, and fast-food consumption per week. To enhance statistical power and address the issue of data sparsity, responses were reclassified into five categories: 0, 1–2, 3–4, 5–6, and 7 or more times per week. A comprehensive dietary score was calculated to provide an integrated assessment of the participants’ overall dietary behavior. While validated dietary assessment tools for Korean adolescents, such as the Nutrient Quotient for Adolescents (NQ-A) [24], exist, they could not be applied in this study because the KYRBS does not collect the necessary information for their calculation. Consequently, a composite dietary behavior score was constructed based on the five dietary behavior items available in the survey. The present scoring system was designed so that a higher total score indicates healthier dietary habits. For breakfast and fruit consumption, the score for each item increased by 1 point with higher frequency. Conversely, for high-caffeine energy drinks, sugar-sweetened beverages, and fast food, the score increased by 1 point as frequency decreased. Consequently, the total score ranged from 0 to 25 points.

### 2.5. Mental Health Characteristics

To assess the mental health characteristics of the participants, GAD and suicidal behaviors, responses to the GAD-7 questionnaire and three suicide-related items from the KYRBS were analyzed [25,26]. For anxiety symptom assessment, individual scores from the seven items of the GAD-7 were summed, resulting in a total score ranging from 0 to 21. Following established clinical cut-offs, participants were classified into two groups: the low-risk group (scores < 10) and the high-risk group (scores ≥ 10) [25,26]. Suicidal behavior variables were dichotomized into yes or no based on whether participants had experienced suicidal ideation, suicide planning, or suicide attempts within the past 12 months. Finally, participants were categorized based on the severity of suicidal behavior. Adolescents who had experienced all three suicidal behaviors were classified as the severe risk group; all others were classified as the low to moderate risk group.

### 2.6. Statistical Analyses

The KYRBS data underwent a thorough cleaning process to address logical errors and outliers, followed by the generation of weights and integration of strata. To accommodate the survey’s complex sampling design, all analyses incorporated the stratification, clustering, and sampling weights provided by the KDCA. We compared the general characteristics, dietary behaviors, and mental health traits of MCA and non-MCA groups using the Rao-Scott chi-square test and complex-sample linear regression, considering the types of variables involved. To assess the association between dietary behavior and mental health issues, we conducted linear and logistic regression analyses, both with and without covariates. For further analysis, we divided the dietary behavior score into tertiles. Results for the tertile groups related to GAD and severe suicidal behavior are presented as odds ratios (ORs) with 95% confidence intervals (CIs), both including and excluding covariates. To evaluate trends in these relationships, we performed additional analyses using the median value of each tertile dietary score as a continuous variable. In the adjusted model, we included covariates such as age, gender, grade, school type, city type, BMI, smartphone usage time, living arrangement, subjective health status, and household economic status. All statistical analyses were conducted using SAS version 9.4 (SAS Institute Inc., Cary, NC, USA), with statistical significance set at *p* < 0.05.

## 3. Results

### 3.1. General Characteristics of MCA and Non-MCA

Table 1 presents the general characteristics of the study participants. Of the 44,796 total participants, approximately 3.83% identified as an MCA, with the majority having a mother of foreign nationality (77.24%). MCA participants exhibited significant differences in various general characteristics compared to non-MCA participants. They were younger (*p* < 0.0001), had a higher BMI (*p* < 0.0001), and had a higher prevalence of residing in county areas (*p* < 0.0001). Additionally, MCAs reported a higher rate of perceived low household economic status (*p* < 0.0001) and a greater proportion enrolled in middle school (*p* = 0.0018). They were also more likely to live separately from family or relatives (*p* < 0.0001) and to perceive their subjective health status as poor (*p* = 0.0020). Furthermore, MCA engaged in longer durations of smartphone usage on both weekdays and weekends (*p* < 0.0001).

### 3.2. Dietary Behaviors and Score of MCA and Non-MCA

Table 2 details the dietary behaviors and behavior scores of MCA and non-MCA participants. MCAs showed a significantly lower weekly frequency of breakfast consumption (*p* = 0.0018) and fruit intake (*p* = 0.0004). No significant difference was found between the two groups in the frequency of high-caffeine energy drinks or sugar-sweetened beverages; however, non-MCA participants consumed fast food more frequently. Additionally, there was no significant difference in the dietary behavior score, an index designed to evaluate overall dietary habits encompassing more than five dietary behaviors.

### 3.3. Mental Health Status of MCA and Non-MCA

Table 3 outlines the mental health-related characteristics of the study participants. No significant differences were found between MCA and non-MCA groups regarding GAD-7 scores or the distribution of the GAD risk group. In terms of suicidal behaviors, there were no significant differences in the distribution of suicidal ideation, suicide planning, or the severe risk group for severe suicidal behavior. However, the proportion of participants who had actually attempted suicide was significantly higher among MCAs compared to non-MCAs (*p* = 0.0107).

### 3.4. Association Between Mental Health and Dietary Behavior of MCA and Non-MCA

The dietary behavior scores based on GAD-7 risk levels and suicidal behavior severity groups are shown in Table 4. In the non-MCA group, participants classified as high-risk for GAD and those in the severe suicidal behavior group exhibited significantly lower dietary behavior scores (all *p* < 0.0001). A similar trend was observed in the MCA group; however, the statistical significance diminished in the adjusted model after accounting for general and socio-environmental factors.

Table 5 presents a further analysis of the relationship between dietary behavior scores and both GAD and the severity of suicidal behavior. In the non-MCA group, the adjusted statistical model revealed that participants in the lower two tertiles of dietary behavior scores were 1.05 (95%CI: 0.97–1.14) and 1.43 (95%CI: 1.33–1.53) times more likely to have GAD compared to those in the highest tertile (*p_trend_* < 0.0001). In the MCA group, the likelihood of having GAD also increased by 1.56 (95%CI: 1.03–2.35) and 1.47 (95%CI: 1.04–2.09) times for those with poorer dietary behaviors (*p_trend_* = 0.0129). However, the interaction between dietary behavior and multicultural background was not statistically significant for GAD. With regard to suicidal behavior, poorer dietary behaviors were also associated with a higher probability of belonging to the severe suicidal behavior group. Compared to the non-MCA group with the highest dietary behavior score, those in the lower two groups exhibited a 24% (95%CI: 0.98–1.58) and 43% (95%CI: 1.16–1.76) increased likelihood of experiencing all three types of suicidal behaviors (*p_trend_* = 0.0013). The relationship between dietary behavior and the severity of suicidal behavior was also highly significant in the MCA group. Individuals with poor dietary habits exhibited a severe tendency towards suicidal behavior that was approximately 14 times higher (95%CI: 2.04–102.7) (*p_trend_* = 0.0023). Moreover, the association between dietary behavior and suicidal behavior severity differed significantly by multicultural family background (*p_interaction_* = 0.0358).

## 4. Discussion

This study utilized the 2024 KYRBS data to examine the dietary behaviors of MCA and non-MCA individuals in South Korea and to identify their associations with mental health issues. The findings revealed that MCA individuals generally had a lower socioeconomic status compared to non-MCA individuals, exhibited certain undesirable dietary behaviors, and demonstrated a significantly higher prevalence of suicide attempts. Additionally, for both MCA and non-MCA groups, poor dietary behaviors were linked to an increased tendency toward GAD and greater severity of suicidal behavior.

Previous studies have indicated that individuals with MCA experience more dietary issues than those without MCAs, which is believed to be linked to their socioeconomic challenges [27,28]. Similar findings were observed in the present study; the frequency of weekly breakfast consumption and fruit intake was significantly lower in the MCA group compared to the non-MCA group. In South Korea, fruit purchase and consumption are influenced by various socioeconomic factors, including gender [29]. For lower-income groups, the high cost of fruit serves as a primary barrier to purchasing and consuming it. Additionally, a study on Korean children found that the frequency of breakfast consumption was higher among children whose parents had higher education levels, held managerial or office positions, or when the mother was a stay-at-home mom [30]. These socioeconomic issues related to fruit and breakfast consumption align with the characteristics of MCAs identified in both previous research and this study. In the present study, a higher proportion of MCAs reported their household economic status as below middle class and lived independently without family or relatives. Additionally, it has been noted that multicultural families, often formed by marriage-immigrant women, frequently face challenges in dietary adaptation [31] and have a relatively higher proportion of temporary or manual labor employment [32].

Although no significant difference was observed in the overall dietary behavior scores between the MCA and non-MCA groups, MCA individuals were less likely to consume breakfast and fruits. Regular breakfast and adequate fruit intake are highly recommended for healthy growth and development [33], as they represent critical dietary behaviors during adolescence—the period when adult eating patterns are established [34]. Therefore, efforts to improve these behaviors, along with fundamental socioeconomic support, are necessary. Interestingly, while fast food consumption is often perceived to be more prevalent among socioeconomically disadvantaged groups or those with poor dietary habits, our study found higher consumption in the non-MCA group. This pattern is consistent with other studies analyzing the dietary behaviors of Korean adolescents, which report that fast food consumption tends to increase with age and higher household economic status [35]. This trend may be related to the specific cultural context of the Korean food environment. In our study, MCA individuals tended to be younger and were more likely to reside in rural areas, away from fast-food restaurants, compared to non-MCA individuals. Furthermore, the price of fast food in Korea is relatively higher than that of alternative snack foods (e.g., instant noodles, gimbap). These general characteristics of MCA individuals and the positioning of fast food within Korean food culture likely contributed to the lower frequency of fast food consumption observed in the MCA group.

The results of this study indicated that there were no significant differences in the distribution of risk groups for GAD and suicidal behaviors between the MCA and non-MCA groups. However, the proportion of individuals who had attempted suicide was significantly higher in the MCA group, highlighting their particular vulnerability to severe mental health issues. Among the three suicide-related behaviors analyzed, the increased prevalence of actual suicide attempts in the MCA group reflects a critical vulnerability in their mental health status. These findings underscore the urgent need for comprehensive support and intervention from both the government and local communities to address these disparities. When examining dietary behaviors in relation to GAD risk and the severity of suicidal behavior, a clear trend emerged: as mental health issues became more severe, dietary behaviors significantly declined. In both the MCA and non-MCA groups, participants with higher GAD risk and severe suicidal behavior scored lower on dietary behaviors. Notably, individuals in the MCA group with severe suicidal behavior had the lowest overall scores. Although this study is limited by its cross-sectional design, the findings support the hypothesis that healthy dietary behaviors may help mitigate mental vulnerability. Previous research has suggested that behaviors such as consuming fruits and vegetables and regularly eating breakfast can reduce the risk of abnormal psychological behaviors by lowering excessive oxidative stress [14,15,16,17,18,19]. The association between dietary behaviors and mental health issues, along with the moderating influence of multicultural family characteristics, was further clarified through logistic regression analysis. When dietary behavior scores were divided into tertiles, a significant dose–response relationship was observed; lower scores were linked to a progressively higher risk of GAD and severe suicidal behavior. The ORs for the association between poor dietary behavior and mental health risks were higher in the MCA group, though caution is warranted in interpreting these values due to their rarity in this population. Additionally, the association between dietary behavior and suicidal behavior severity appeared to differ by multicultural family background.

The increasing proportion of MCAs highlights their growing significance in national policy, particularly given that South Korea’s fertility rate is one of the lowest in the world [9]. As mentioned earlier, adolescents raised in diverse nationalities and cultures may experience identity confusion stemming from their multicultural environment, in addition to the typical conflicts associated with life stages. This added complexity can negatively impact their mental health [10,11]. Although recent government reports indicate improvements in the economic status of multicultural families—such as increases in median income and higher education enrollment rates for their children—they still face significant challenges compared to the mainstream population [32]. Furthermore, since most multicultural families are formed through marriage to immigrant women, there is an inherent potential for difficulties in dietary acculturation and subsequent nutritional issues [31,32]. The findings of this study suggest that dietary behaviors and quality are associated with the mental health of Korean adolescents and underscore the urgent need for increased attention and support for an MCA as a vulnerable population.

This study suggested the relationship between dietary behaviors and mental health among MCAs in South Korea, utilizing large-scale, representative data from the 2024 KYRBS. Despite its strengths, the study has several limitations. First, as a cross-sectional study, the KYRBS has inherent limitations in establishing causal relationships between dietary behaviors and mental health outcomes. Second, the lack of detailed information on specific food items and nutrient intake in the survey limits a more comprehensive analysis of dietary quality. This structural constraint also precluded the application of existing validated dietary indices, such as the NQ-A. As a result, the composite dietary behavior score in this study was created solely from the five behavioral frequency items available in the KYRBS, without formal validation or differential weighting. While this score offers a practical summary of overall dietary behavior patterns, its limitations must be acknowledged, and findings related to this score should be interpreted with caution. Additionally, the relatively small proportion of MCAs restricts the statistical precision of subgroup analyses, including those stratified by parental nationality and those examining severe suicidal behavior, where the number of subjects in each subgroup is very limited. Therefore, readers are encouraged to focus on the overall directional trends indicated by the analyses, and these findings should be viewed as exploratory, pending validation in larger samples. Lastly, since the KYRBS relies on subjective, self-reported online responses, the clinical accuracy regarding GAD and overall mental health status may be limited. Therefore, caution is advised when interpreting the findings.

## 5. Conclusions

The study found that MCAs experience poorer dietary behaviors and socioeconomic conditions compared to their non-MCA peers, along with a higher prevalence of suicide attempts. Additionally, regardless of multicultural background, poorer dietary behaviors were linked to an increased likelihood of GAD and higher severity of suicidal behavior, with this association being particularly pronounced in MCA. These findings indicate that comprehensive and multifaceted policy support from both local communities and the government is crucial for improving the dietary and mental health status of MCA in Korea.

## Figures and Tables

**Table 1:** General characteristics of study subjects, taking into account multicultural backgrounds.

		All	Non-MCA	MCA	*p*
Number of subjects		44,796 (100.0)	43,079 (96.16)	1717 (3.83)	<0.0001
Age (years)		14.9 ± 0.02	14.9 ± 0.02	14.6 ± 0.04	<0.0001
Gender	Boys	22,349 (49.97)	21,509 (49.99)	840 (49.5)	0.7645
	Girls	22,447 (50.02)	21,570 (50.01)	877 (50.49)	
Body mass index (kg/m^2^)		21.39 ± 0.03	21.30 ± 0.03	21.70 ± 0.09	<0.0001
Living area	County	2444 (4.15)	2220 (3.94)	224 (10.23)	<0.0001
	City	19,974 (46.46)	19,135 (46.36)	839 (48.97)	
	Metropolitan city	22,378 (49.39)	21,724 (49.69)	654 (40.78)	
School type	High	20,466 (48.26)	19,807 (48.47)	659 (42.3)	0.0018
	Middle	24,330 (51.73)	23,272 (51.52)	1058 (57.6)	
School year		3.41 (0.02)	3.4 (0.02)	3.1 (0.04)	<0.0001
Household income	High	5228 (11.81)	5125 (12.01)	103 (6.32)	<0.0001
	Mid-high	14,443 (32.85)	14,116 (33.3)	327 (19.34)	
	Mid-low	20,760 (45.99)	19,859 (45.77)	901 (52.38)	
	Low	4365 (9.33)	3979 (8.89)	386 (21.94)	
Living arrangement	Cohabitation	43,184 (97.14)	41,574 (97.23)	1610 (94.56)	<0.0001
	Alone	1612 (2.85)	1505 (2.76)	107 (5.43)	
Subjective health status	Very healthy	9790 (21.71)	9452 (21.78)	338 (19.81)	0.0020
	Healthy	20,206 (45.13)	19,473 (45.23)	733 (42.39)	
	Average	10,758 (24.12)	10,292 (23.99)	466 (27.83)	
	Unhealthy	4042 (9.02)	3862 (8.98)	180 (9.95)	
Multicultural family type	Both Korean parents	43,079 (96.63)			
	International mother	1384 (2.6)		1384 (77.24)	
	International father	88 (0.21)		88 (6.11)	
	Both international parents	245 (0.56)		245 (16.65)	
Smart mobile use (weekdays)	No	996 (2.14)	969 (2.15)	27 (1.88)	0.5164
	Yes	43,800 (97.85)	42,110 (97.84)	1690 (98.11)	
	Time (minutes)	261.5 ± 1.58	259.9 ± 1.57	305.1 ± 4.76	<0.0001
Smart mobile use (weekends)	No	990 (2.29)	964 (2.3)	26 (1.71)	0.1373
	Yes	43,806 (97.71)	42,115 (97.69)	1691 (98.29)	
	Time (minutes)	378.3 ± 2.17	375.3 ± 2.16	463.3 ± 6.25	<0.0001

MCA, adolescents from multicultural families. Numbers are mean and standard error for age, school year, body mass index and mobile use time, otherwise number of subjects (%). *p* values for age, school year, body mass index and mobile use time were obtained from logistic regression analyses between non MF and MF, otherwise Rao-Scott χ^2^ tests.

**Table 2:** Dietary behavior (frequency/week) of the study population, considering family type.

	All	Non-MCA	MCA	*p*
Breakfast consumption				
0	11,002 (24.65)	10,547 (24.56)	455 (27.26)	0.0018
1–2	7581 (16.92)	7268 (16.88)	313 (18.17)	
3–4	6182 (13.50)	5919 (13.45)	263 (15.06)	
4–5	7996 (17.76)	7717 (17.81)	279 (16.29)	
7	12,035 (27.15)	11,628 (27.28)	407 (23.20)	
Fruits consumption				
0	4654 (10.40)	4440 (10.31)	214 (12.94)	0.0004
1–2	14,092 (31.18)	13,518 (31.13)	574 (32.62)	
3–4	12,810 (28.44)	12,319 (28.43)	491 (28.62)	
4–5	4848 (10.94)	4670 (10.96)	178 (10.32)	
≥7	8392 (19.01)	8132 (19.14)	260 (15.47)	
Caffeine drink consumption				
0	22,916 (50.21)	22,027 (50.21)	889 (50.24)	0.4474
1–2	11,969 (26.79)	11,489 (26.77)	480 (27.35)	
3–4	5146 (11.81)	4979 (11.86)	167 (10.54)	
4–5	1925 (4.48)	1839 (4.45)	86 (5.22)	
≥7	2840 (6.68)	2745 (6.68)	95 (6.63)	
Sweetened beverage consumption				
0	2594 (5.76)	2484 (5.75)	110 (6.07)	0.1021
1–2	13,576 (30.02)	13,014 (29.93)	562 (32.60)	
3–4	15,005 (33.52)	14,468 (33.61)	537 (30.89)	
4–5	6590 (14.80)	6360 (14.83)	230 (13.73)	
≥7	7031 (15.88)	6753 (15.86)	278 (16.68)	
Fast foods consumption				<0.0001
0	6826 (15.00)	6479 (14.83)	347 (20.0)	
1–2	25,548 (56.70)	24,586 (56.77)	962 (54.81)	
3–4	9877 (22.40)	9561 (22.50)	316 (19.56)	
4–5	1631 (3.81)	1572 (3.81)	59 (3.63)	
≥7	914 (2.06)	881 (2.06)	33 (1.95)	
Dietary behavior score	16.91 ± 0.01	16.91 ± 0.01	17.01 ± 0.08	0.2312

MCA, adolescents from multicultural families. Data are presented as mean and standard error for dietary behavior score, otherwise as number of subjects (%). *p* value for dietary behavior score is from adjusted regression model with age, gender, school year and type, body mass index, mobile phone using time, city type, cohabitation type, health status, and household income, otherwise from Rao-Scott χ^2^ tests.

**Table 3:** Mental health issues—GAD and suicidal behavior—taking into account family type.

	All	Non-MCA	MCA	*p*
GAD-7 score	4.51 ± 0.03	4.51 ± 0.03	4.41 ± 0.11	0.4204
GAD				
Low risk	38,523 (85.90)	37,038 (85.90)	1485 (85.89)	0.9952
High risk	6273 (14.09)	6041 (14.09)	232 (14.10)	
Suicidal ideation				
No	39,226 (87.57)	37,734 (87.58)	1492 (87.20)	0.6616
Yes	5570 (12.42)	5345 (12.41)	225 (12.79)	
Suicide planning				
No	42,755 (95.49)	41,121 (95.49)	1634 (95.32)	0.7454
Yes	2041 (4.50)	1958 (4.50)	83 (4.67)	
Suicide attempts				
No	43,674 (97.56)	42,018 (97.60)	1656 (96.40)	0.0107
Yes	1122 (2.43)	1061 (2.39)	61 (3.59)	
Severe suicidal behavior				
Low-moderate risk	44,148 (98.59)	42,461 (98.60)	1687 (98.33)	0.7003
Severe risk	648 (1.40)	618 (1.39)	30 (1.66)	

GAD-7, Generalized Anxiety Disorder 7-item scale; MCA, adolescents from multicultural families. Data are presented as mean and standard error for the GAD-7 score; otherwise, they are presented as the number of subjects (%). *p* value for GAD-7 score was obtained from regression analyses between non-MCA and MCA; otherwise, Rao-Scott χ^2^ tests were used.

**Table 4:** Dietary behavior score of study subjects, taking into account GAD and suicidal severity groups.

		Non-MCA				MCA			
		N	Mean	*p_crude_*	*p_adjusted_*	N	Mean	*p_crude_*	*p_adjusted_*
GAD	Low risk	37,038	17.01 ± 0.01	<0.0001	<0.0001	1485	17.11 ± 0.08	0.0004	0.0270
	High risk	6041	16.30 ± 0.03			232	16.39 ± 0.18		
Severe suicidal behavior	Low-moderate risk	42,461	16.93 ± 0.01	<0.0001	<0.0001	1687	17.04 ± 0.08	0.0064	0.0522
	Severe risk	618	15.93 ± 0.12			30	15.07 ± 0.71		

GAD, Generalized Anxiety Disorder; MCA, adolescents from multicultural families; N, number of subjects. Data are presented as mean and standard error. *p_crude_* refers to crude regression models between the two groups in non-MCA and MCA. *p_adjusted_* refers to the adjusted regression model accounting for age, gender, school year and type, body mass index, mobile phone usage time, city type, cohabitation type, health status, and household income.

**Table 5:** Association between dietary behavior score and GAD and severe suicidal behavior.

**GAD**	**Low Risk** **(*n*, %)**	**Mean Dietary Behavior Score for Each Tertile**	**High Risk (*n*, %)**	**Mean Dietary Behavior Score for Each Tertile**	**OR*_crude_* (95%CI)**	**OR*_adjusted_* (95%CI)**	***p_trend_***	***p_interaction_***
Non-MCA								
1st tertile	14,709 (40.49)	14.20 ± 0.01	3034 (50.72)	13.86 ± 0.01	1.57 (1.47–1.68)	1.43 (1.33–1.53)	<0.0001	
2nd tertile	10,269 (27.48)	17.49 ± 0.00	1456 (23.84)	17.46 ± 0.01	1.09 (1.01–1.18)	1.05 (0.97–1.14)		
3rd tertile	12,060 (32.02)	20.16 ± 0.01	1551 (25.43)	20.08 ± 0.01	Ref	Ref		
Total	37,038 (100.0)	17.01 ± 0.01	6041 (100.0)	16.30 ± 0.03				
MCA								0.0807
1st tertile	558 (38.41)	14.16 ± 0.08	104 (47.65)	13.82 ± 0.19	1.72 (1.25–2.38)	1.47 (1.04–2.09)	0.0129	
2nd tertile	401 (24.73)	17.51 ± 0.02	69 (28.56)	17.48 ± 0.05	1.61 (1.10–2.36)	1.56 (1.03–2.35)		
3rd tertile	526 (36.84)	20.25 ± 0.06	59 (23.78)	20.22 ± 0.16	Ref	Ref		
Total	1485 (100.0)	17.11 ± 0.08	232 (100.0)	16.39 ± 0.18				
Severe suicidal behavior	Low-moderate risk (*n*, %)	Mean dietary behavior score for each tertile	Severe risk (*n*, %)	Mean dietary behavior score for each tertile	OR*_crude_* (95%CI)	OR*_adjusted_* (95%CI)	*p_trend_*	*p_interaction_*
Non-MCA								
1st tertile	17,414 (41.77)	14.15 ± 0.01	329 (53.43)	13.45 ± 0.13	1.81 (1.50–2.18)	1.43 (1.16–1.76)	0.0013	
2nd tertile	11,572 (27.01)	17.48 ± 0.00	153 (24.54)	17.48 ± 0.04	1.28 (1.02–1.62)	1.24 (0.98–1.58)		
3rd tertile	13,475 (31.22)	20.15 ± 0.01	136 (22.02)	20.20 ± 0.10	Ref	Ref		
Total	42,461 (100.0)	16.93 ± 0.01	618 (100.0)	15.93 ± 0.12				
MCA								0.0358
1st tertile	644 (40.69)	14.13 ± 0.07	18 (63.03)	13.28 ± 0.71	12.12 (2.94–49.84)	14.48 (2.04–102.7)	0.0023	
2nd tertile	460 (25.85)	17.50 ± 0.02	10 (32.69)	17.69 ± 0.13	9.89 (2.25–43.50)	14.95 (2.02–110.4)		
3rd tertile	583 (33.44)	20.24 ± 0.05	2 (4.27)	21.60 ± 1.11	Ref	Ref		
Total	1687 (100.0)	17.04 ± 0.08	30 (100.0)	15.07 ± 0.71				

GAD, Generalized Anxiety Disorder; MCA, adolescents from multicultural families; *n*, number of subjects; OR, odds ratio; Ref, reference; 95%CI, 95% confidence interval. Data are presented as the mean and standard error for the dietary behavior score for each tertile; otherwise, the number of subjects (%) is provided. OR*_crude_* values are from crude logistic regression models. OR*_adjusted_* values are from an adjusted logistic regression model that includes age, gender, school year and type, body mass index, mobile phone usage time, city type, cohabitation type, health status, and household income. *p_interaction_* values are derived from the interaction between the dietary score and the multicultural family type in the GAD group and suicidal severity group phenotype.

## Data Availability

This study analyzed data from the 2024 KYRBS. The data can be downloaded from the KYRBS official website: https://www.kdca.go.kr/yhs/ (accessed on 2 March 2026).

## Abbreviations

BMI	body mass index
CIs	95% confidence intervals
GAD	generalized anxiety disorder
IRB	Institutional Review Board
KYRBS	Korea Youth Risk Behavior Survey
MCA	adolescents from multicultural families
NQ-A	Nutrient Quotient for Adolescents
ORs	odds ratios

## References

[1] Petanjek Z., Judaš M., Šimic G., Rasin M.R., Uylings H.B., Rakic P., Kostovic I. (2011). Extraordinary neoteny of synaptic spines in the human prefrontal cortex. Proc. Natl. Acad. Sci. USA.

[2] Conte G., Iorio G.D., Esposito D., Romano S., Panvino F., Maggi S., Altomonte B., Casini M.P., Ferrara M., Terrinoni A. (2025). Scrolling through adolescence: A systematic review of the impact of TikTok on adolescent mental health. Eur. Child Adolesc. Psychiatry.

[3] World Health Organization Mental Health of Adolescents. https://www.who.int/news-room/fact-sheets/detail/adolescent-mental-health.

[4] Benton T.D., Muhrer E., Jones J.D., Lewis J. (2021). Dysregulation and Suicide in Children and Adolescents. Child Adolesc. Psychiatr. Clin. N. Am..

[5] Statistics Korea (2024). Statistics on Children and Youth.

[6] Choi J.W., Moon H.Y., Jeon J.A., Park Y.C. (2021). Survey on the Mental Health of Teenagers.

[7] Statistics Korea (2024). 2023 Vital Statistics of Multicultural Families.

[8] Ministry of the Interior and Safety (2024). 2023 Census of Foreign Residents in Local Governments.

[9] Kim J.K. (2025). The predetermined future: Tackling South Korea’s total fertility rate crisis. Clin. Exp. Pediatr..

[10] Kim M.-K. (2019). A Convergent Study of Variables Influencing on Suicide Ideation of Adolescents in Multicultural Family. J. Korea Converg. Soc..

[11] Jang I. (2020). Comparative Study of Health Risk Behaviors, Mental Health and Subjective Health Status of Adolescents in Multicultural and Monocultural Families. J. Korean Soc. Sch. Health.

[12] Lee S.J., Ryu H.K. (2023). A Study of Depression Related Factors and Their Association with Diet in People in Young-, Middle-, and Late Adulthood-Based on Data from the Korea National Health and Nutrition Examination Survey, 2016 and 2018. J. Korean Soc. Food Sci. Nutr..

[13] Yoon Y.S., Oh S.W. (2021). Relationship between psychological distress and the adherence to the Korean healthy eating index (KHEI): The Korea National Health and Nutrition Examination Survey (KNHANES) 2013 and 2015. Nutr. Res. Pract..

[14] Malmir H., Mahdavi F.S., Ejtahed H.S., Kazemian E., Chaharrahi A., Mohammadian Khonsari N., Mahdavi-Gorabi A., Qorbani M. (2023). Junk food consumption and psychological distress in children and adolescents: A systematic review and meta-analysis. Nutr. Neurosci..

[15] Knüppel A., Shipley M.J., Llewellyn C.H., Brunner E.J. (2017). Sugar intake from sweet food and beverages, common mental disorder and depression: Prospective findings from the Whitehall II study. Sci. Rep..

[16] Molendijk M., Molero P., Ortuño Sánchez-Pedreño F., Van der Does W., Angel Martínez-González M. (2018). Diet quality and depression risk: A systematic review and dose-response meta-analysis of prospective studies. J. Affect. Disord..

[17] Matison A.P., Flood V.M., Lam B.C.P., Lipnicki D.M., Tucker K.L., Preux P.M., Guerchet M., d’Orsi E., Quialheiro A., Rech C.R. (2024). Associations between fruit and vegetable intakes and incident depression in middle-aged and older adults from 10 diverse international longitudinal cohorts. J. Affect. Disord..

[18] Lu X., Wu L., Shao L., Fan Y., Pei Y., Lu X., Borné Y., Ke C. (2024). Adherence to the EAT-Lancet diet and incident depression and anxiety. Nat. Commun..

[19] Zhao Y., Chen M., Wang J., Ye Z., Qu Y., Wang Z., Wang X., Jiang Y. (2025). Association of Diet Quality with Depression, Anxiety, and Comorbidity Symptoms in Chinese School-Aged Children. Nutrients.

[20] Choi Y.-S. (2015). Comparison of Dietary Habits of Adolescents from Multicultural and General Korean Families: The 9th Korea Youth Risk Behavior Web-based Survey. Korean Parent-Child Health J..

[21] Kim S., Choi J.-H., Heo Y.-R. (2025). Fruit Consumption and Mental Health in Adolescents from Multicultural and Non-multicultural Families: Data from Korea Youth Risk Behavior Web Based Survey 2021. Hum. Ecol. Res..

[22] Song S. (2020). Comparison of Dietary and Lifestyle Behavior and Weight Status among Adolescents from Multicultural Families and Non-Multicultural Families: Based on the 2017–2018 Korea Youth Risk Behavior Surveys Data. Korean J. Hum. Ecol..

[23] Oh J., Chung J. (2020). Fruit and Vegetable Consumption Frequency and Mental Health in Korean Adolescents: Based on the 2014–2017 Korea Youth Risk Behavior Survey. J. Nutr. Health.

[24] Kim H.Y., Lee J.S., Hwang J.Y., Kwon S., Chung H.R., Kwak T.K., Kang M.H., Choi Y.S. (2017). Development of NQ-A, Nutrition Quotient for Korean Adolescents, to assess dietary quality and food behavior. J. Nutr. Health.

[25] Spitzer R.L., Kroenke K., Williams J.B., Löwe B. (2006). A brief measure for assessing generalized anxiety disorder: The GAD-7. Arch. Intern. Med..

[26] Ahn J.K., Kim Y., Choi K.H. (2019). The Psychometric Properties and Clinical Utility of the Korean Version of GAD-7 and GAD-2. Front. Psychiatry.

[27] Song S., Song H. (2019). Dietary and Lifestyle Factors Associated with Weight Status among Korean Adolescents from Multicultural Families: Using Data from the 2017–2018 Korea Youth Risk Behavior Surveys. Korean J. Community Nutr..

[28] Hu Y.R., Song S. (2022). Dietary Behaviors Associated with Health Perception of Korean Adolescents from Multicultural Families: Based on data from the 2017~2019 Korea Youth Risk Behavior Surveys. Korean J. Community Nutr..

[29] Kim H., Seo S. (2014). Factors influencing on intention to intake fruit: Moderating effect of fruit intake habit. J. Nutr. Health.

[30] Yu S.Y., Yang Y.J. (2019). Nutritional status and related parental factors according to the breakfast frequency of elementary school students: Based on the 2013~2015 Korea National Health and Nutrition Examination Survey. J. Nutr. Health.

[31] Kim J.-M., Lee H.S., Kim M.H. (2012). Food adaptation and nutrient intake of female immigrants into Korea through marriage. Korean J. Nutr..

[32] Ministry of Gender Equality and Family (2025). 2024 National Survey of Multicultural Families.

[33] Rampersaud G.C., Pereira M.A., Girard B.L., Adams J., Metzl J.D. (2005). Breakfast habits, nutritional status, body weight, and academic performance in children and adolescents. J. Am. Diet. Assoc..

[34] Brug J. (2008). Determinants of healthy eating: Motivation, abilities and environmental opportunities. Fam. Pract..

[35] Hong S.H. (2022). Factors Influencing Fast Food Consumption in Korean Adolescents—Based on the 16th Korea Youth Risk Behavior Web-based Survey. Korean J. Food Nutr..

